# Layer-specific cellular composition of mouse primary somatosensory and human temporal cortex: a direct 3D confocal counting approach

**DOI:** 10.3389/fnana.2026.1795807

**Published:** 2026-04-23

**Authors:** Sergio Plaza-Alonso, Lidia Alonso-Nanclares, Silvia Tapia-González, Laura Fernández-García, Asta Kastanauskaite, Javier DeFelipe

**Affiliations:** 1 Laboratorio Cajal de Circuitos Corticales (CTB), Universidad Politécnica de Madrid, Madrid, Spain; 2 Cajal Neuroscience Center, CSIC, Madrid, Spain; 3 Departamento de Ciencias Médicas Básicas, Facultad de Medicina-Instituto de Medicina Molecular Aplicada-Nemesio Díez (IMMA-ND), Universidad San Pablo-CEU, CEU Universities, Madrid, Spain

**Keywords:** cell counting, cortical columns, glia number, glia-to-neuron ratio, neuron number

## Abstract

Accurate quantification of cellular composition is fundamental to understanding the structural and functional organization of the cerebral cortex. In the present study, we quantified the proportions of neurons, glia, and vascular cells (primarily endothelial cells) across the full cortical thickness (layers I–VI) of the mouse primary somatosensory cortex (S1HL) and the human temporal cortex (BA21) using immunocytochemical techniques and a direct 3D counting method. Over 25,000 cells in the mouse and 13,000 cells in the human cortex were individually identified and classified. For this purpose, we utilized EspINA software, which enables precise cell identification and volumetric analysis while preserving laminar and spatial context. Our results reveal marked species-specific differences in cellular proportions: neurons represent approximately 60% of all cells in the mouse S1HL but only 30% in the human BA21. These differences are reflected in the glia-to-neuron ratio (GNR) and non-neuron-to-neuron ratio (nNNR), which were consistently below 1.0 in the mouse (GNR: 0.4; nNNR: 0.6) but significantly higher in the human samples (GNR: 1.5; nNNR: 2.3). By overcoming the limitations of traditional stereological and tissue-homogenization techniques, this study provides a detailed laminar characterization of the cellular composition in these particular cortical regions (mouse S1HL and human BA21).

## 1 Introduction

Accurate quantification of neurons, glia, and vascular cells is fundamental to understanding cortical organization, yet remains technically challenging across species (DeFelipe, 2015). While extensive methodological advances exist (Schmitz et al., 2014; von Bartheld et al., 2016), most studies rely on stereology or tissue homogenization, which sacrifice laminar resolution or introduce cell-type biases. Three main methodologies for brain cell counting have been developed (reviewed in von Bartheld et al., 2016): (1) Direct counting: Originally developed in the 1950s, this method counts cells by collecting nuclei suspended in a homogenized volume of brain tissue (Nurnberger and Gordon, 1957; Brizzee et al., 1964). It later evolved into the isotropic fractionator (IF) method, which has been applied to rodent, primate, and human brains (Herculano-Houzel and Lent, 2005; Azevedo et al., 2009; Herculano-Houzel et al., 2006, 2015); (2) DNA extraction and quantitation: Also introduced in the 1950s, this method estimates cell numbers by measuring DNA content, based on the known amount of DNA per cell nucleus (Nurnberger and Gordon, 1957; Johnson and Erner, 1972; Hess and Thalheimer, 1971); and (3) Histological methods: These widely used approaches estimate brain cell numbers by examining stained tissue sections under a microscope. Among histological techniques, stereology—a design-based approach—has been widely adopted due to its capacity to estimate cell numbers using thick sections and systematic random sampling (reviewed in West, 2012). The method introduced by Sterio (1984) provides unbiased estimates of cell numbers in a three-dimensional volume of brain tissue and is widely regarded as the gold standard for cell quantification in the human brain (Pakkenberg and Gundersen, 1997; West and Gundersen, 1990) and in other species (Haug, 1987; Beaulieu and Colonnier, 1989; Stolzenburg et al., 1989; Beaulieu, 1993; Skoglund et al., 1996).

More recently, a variety of automated detection software tools have been developed to count cells in brain sections (reviewed in Meijering et al., 2012; Schmitz et al., 2014; Toyoshima et al., 2016; LaTorre et al., 2021). These automated tools offer significant practical advantages, including improved reproducibility and the elimination of time-consuming manual counting. Despite the availability of numerous methods and analyses developed to estimate brain cell numbers, discrepancies frequently arise between reported results, and precise knowledge of the total number of brain cells remains elusive because of methodological and technical challenges.

Some of these differences can be attributed to the diverse methodologies and approaches used for cell counting (Sterio, 1984; Korbo et al., 1990; Beaulieu et al., 1992; Beaulieu, 1993; reviewed in von Bartheld et al., 2016). In particular, estimates of cell numbers may vary depending on tissue-processing protocols (fixation, sectioning, staining methods, etc.) as well as biological variability, which can introduce bias into all counting methods. Furthermore, some of the above-mentioned methods do not accurately delineate brain regions, which can be an important source of discrepancy in cell-number estimates. This limitation is especially relevant for the mammalian neocortex, a multilaminar structure with substantial cytoarchitectonic variation between cortical areas and cortical layers.

As reviewed by Herculano-Houzel (2014), analyses of the glia-to-neuron ratio have revealed considerable variation across species and brain regions, and this ratio is often used in comparative studies because it reflects how much glial support is available per neuron and how this relationship scales with neuronal density and size. However, given the wide range of reported ratios, it is difficult to obtain a clear idea of the actual proportions of glial and neuronal cells across species, brain regions, and subregions.

Comparisons between humans and experimental animals are particularly difficult to interpret since brain fixation in experimental animals is usually performed by perfusion, whereas human brains are obtained at autopsy by immersion in the fixative after a postmortem delay that can range from hours to days or even weeks. This delay may have important consequences at multiple levels of biological organization, leading to genetic, molecular, biochemical, and anatomical changes, as shown by our group and others (Gonzalez-Riano et al., 2017; Merino-Serrais et al., 2020, and references therein).

Thus, in the present study, we applied a direct cell-counting method to accurately determine the proportions of neurons, glia, and vascular cells (endothelial cells lining blood vessels as well as associated pericytes and smooth muscle cells) across the full cortical thickness (layers I–VI) of the mouse primary somatosensory cortex and the human temporal cortex under different fixation conditions. These represent non-homologous isocortical regions (primary somatosensory in mouse and temporal association in human), selected to validate the counting approach across diverse cytoarchitectures under different fixation conditions. Furthermore, the present study leverages our existing high-quality datasets from these cortical regions for future integrative modeling efforts (e.g., Kanari et al., 2025). Although direct counting is extremely time-consuming, it enables precise identification and quantification, since each cell nucleus within a given volume of brain tissue can be individually tagged and unequivocally classified as neuronal or non-neuronal using specific labeling. The values obtained using this method are highly informative even though only a relatively small portion of the cortex is analyzed, and they serve as a proof of concept for estimating the proportions of neurons versus other non-neuronal cells. In the present study, more than 25,000 cells from the mouse cortex and 13,000 cells in the human cerebral cortex were individually identified and classified according to their cell type. It should also be noted that the comparison between the human and mouse data in this study is not intended to support functional or evolutionary inferences, but simply to illustrate differences in cellular composition between two distinct cortical regions.

## 2 Materials and methods

### 2.1 Tissue preparation

Two-month-old male C57BL/6J mice (*n* = 6) were sacrificed by administration of a lethal intraperitoneal injection of sodium pentobarbital (40 mg/kg). Two mice were intracardially perfused with 4% paraformaldehyde in 0.1 M phosphate buffer (PB). Their brains were extracted from the skull and fixed overnight in the same fixative at 4°C. The other four mice were sacrificed with the same dose of pentobarbital; their brains were removed from the skull after postmortem time (PMT) intervals of 0 h and 5 h (*n* = 2 animals per interval), as described in Gonzalez-Riano et al. (2017), and subsequently fixed by immersion in the same fixative at 4°C. Animals were handled in accordance with the European Community Directive 2010/63/EU, and all procedures were approved by the local ethics committee of the Spanish National Research Council (CSIC).

Brains from all six animals were rinsed in PB and cut into 50-μm-thick coronal slices using a vibratome (Leica VT2100S, St. Louis, MO), and collected serially. Sections containing the hindlimb region of the primary somatosensory cortex (S1HL; Bregma -070/-1.22 mm, Interaural 3.10/2.58 mm; Paxinos and Franklin, 2001) were selected for immunostaining and cell counting.

Human brain tissue was obtained at autopsy from the Unidad Asociada Neuromax (Laboratorio de Neuroanatomía Humana, Facultad de Medicina, Universidad de Castilla-La Mancha, Albacete), following national laws and international ethical guidelines for the use of human samples in biomedical research. We used samples from three control brains (no recorded neurological or psychiatric alterations): two males aged 53 (AB3) and 66 (AB7) years, and one female aged 53 years (AB2). PMT between death and fixation ranged from 2.5 to 4 h (Table 1). Tissue samples from the same cortical region and individuals have been used in prior studies showing excellent preservation (Cano-Astorga et al., 2021, 2023; Tapia-González and DeFelipe, 2023).

**TABLE 1:** Clinical and neuropsychological information from the human cases analyzed.

Case	Sex	Age (years)	Cause of death	PMT (h)	Neurological diagnosis
AB2	Female	53	Pulmonary shock	4	No neurological alterations
AB3	Male	53	Metastatic bladder carcinoma	3.5	No neurological alterations
AB7	Male	66	Metastatic bladder carcinoma	2.5	No neurological alterations

PMT, Postmortem time between death and fixation.

Upon removal, brains (AB2, AB3, AB7) were immediately fixed in cold 4% paraformaldehyde in 0.1 M PB (pH 7.4) and sectioned into ∼1.5 cm-thick coronal slabs. The temporal cortical area corresponding to Brodmann area 21 at a distance of 2–3 cm from the temporal pole (BA21; Amunts and Zilles, 2015) was cut into ∼1 × 1 × 1 cm blocks and post-fixed in the same fixative for 24–48 h at 4°C. Blocks were cryoprotected in 25% sucrose in PB and stored at -20°C in a solution of glycerol, ethylene glycol, and PB. Serial 50-μm sections of each case were obtained using a vibratome (Leica VT2100S, St. Louis, MO) and processed for immunostaining.

### 2.2 Immunostaining

Free-floating sections were incubated for 2 h in blocking solution containing 3% normal goat serum (Vector Laboratories, Burlingame, CA, United States) in 0.1 M phosphate buffer (PB) with 0.5% Triton X-100. Sections were then incubated overnight at 4°C in the same solution containing primary antibodies: (i) mouse sections with a mixture of rabbit anti-neuron-specific nuclear protein (NeuN; 1:2,000; Chemicon, Temecula, CA, United States) and mouse anti-endothelial cell (RECA-1; 1:2,000; Abcam plc, Cambridge, United Kingdom); (ii) human sections with rabbit anti-NeuN (1:2,000; Chemicon).

After rinsing in PB, the sections were incubated for 2 h at room temperature with appropriate fluorophore-conjugated secondary antibodies: Alexa Fluor 594 goat anti-rabbit (1:1,000; Molecular Probes, Eugene, OR, United States) and Alexa Fluor 647 goat anti-mouse (1:1,000; Molecular Probes, Eugene, OR, United States). Thereafter, all sections were incubated with a 10 μM solution of the fluorescent dye 4’,6-diamidino-2-phenylindole (DAPI; Sigma D9542, St. Louis, MO, United States) for nuclear labeling.

To eliminate lipofuscin autofluorescence in human samples, Autofluorescence Eliminator Reagent (2160; Millipore, Burlington, MA, United States) was applied following the manufacturer’s instructions. After staining, all sections were mounted and coverslipped with ProLong Gold Antifade Reagent (Invitrogen, Carlsbad, CA, United States).

### 2.3 Image acquisition

Sections were examined using a Zeiss 710 confocal laser scanning system (Carl Zeiss Microscopy GmbH, Jena, Germany). In mouse sections, fluorescence signals for neurons (NeuN-immunoreactivity: NeuN-ir), blood vessels (RECA-1-immunoreactivity: RECA-1-ir), and cell nuclei (DAPI staining) were recorded through three separate channels. In human sections, NeuN-ir and DAPI signals were recorded through two channels.

Confocal image stacks were obtained with a 40 × oil-immersion lens (N.A. 1.3) for mouse samples and a 20 × objective lens (N.A. 0.8) for human sections, using a z-step of 1 μm (yielding ∼40–70 z-planes) and a scanning resolution of 1,024 × 1,024 pixels. Image resolution in the xy-plane was 0.83 μm/pixel for mouse stacks and 0.69 μm/pixel for human stacks. Acquisition of final images spanning all cortical layers was performed using the “Tile Scan” tool with 10% overlap, followed by stitching in ZEN software (2012, SP1 v8.1; Carl Zeiss Microscopy). Final images comprised ∼16–30 tiles across all channels. Throughout this manuscript, the term “cortical column” refers to this composite stitched image from the cortical surface to the gray/white matter interface. Cortical layers in both human and mouse samples were identified based on the density, size and shape of neuronal bodies, labeled with NeuN.

**FIGURE 1:**
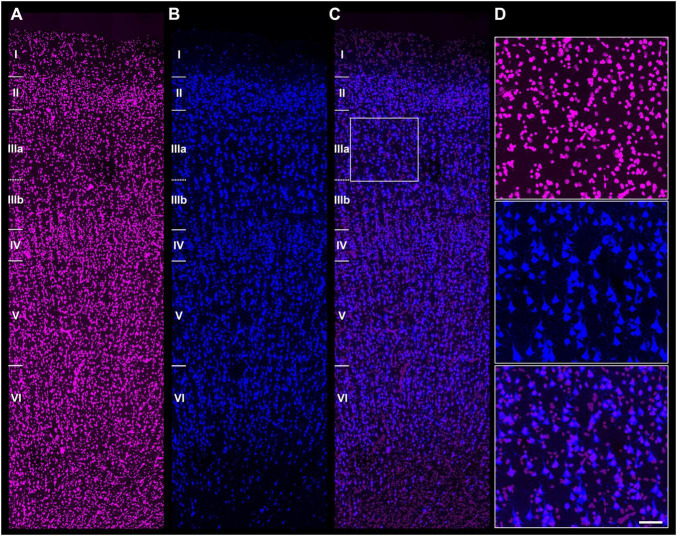
Double labeled section from the human BA 21. **(A)** Section labeled with DAPI (in purple). Cortical layers I–VI are indicated. **(B)** Same section as in panel **(A)**, labeled with NeuN (in blue). **(C)** Composite image obtained by combining **(A,B)**. **(D)** Higher magnification images from (**A,** top), (**B**, middle), and (**C,** bottom) showing the colocalization of DAPI nuclei and NeuN-ir neurons. Scale [in panel **D**)]: 160 μm in panels **(A–C)**; 60 μm in panel **(D)**.

For the mouse brain studies, 14 image stacks containing complete S1HL cortical columns were acquired: 6 from perfusion-fixed brains (2 animals) and 8 from immersion-fixed brains (4 animals). For the human temporal cortex analysis, 6 stacks containing complete BA21 cortical columns were acquired. An example of a double-labeled human brain section (DAPI/NeuN) confocal stack is shown in Figure 1.

### 2.4 Cell segmentation

**FIGURE 2:**
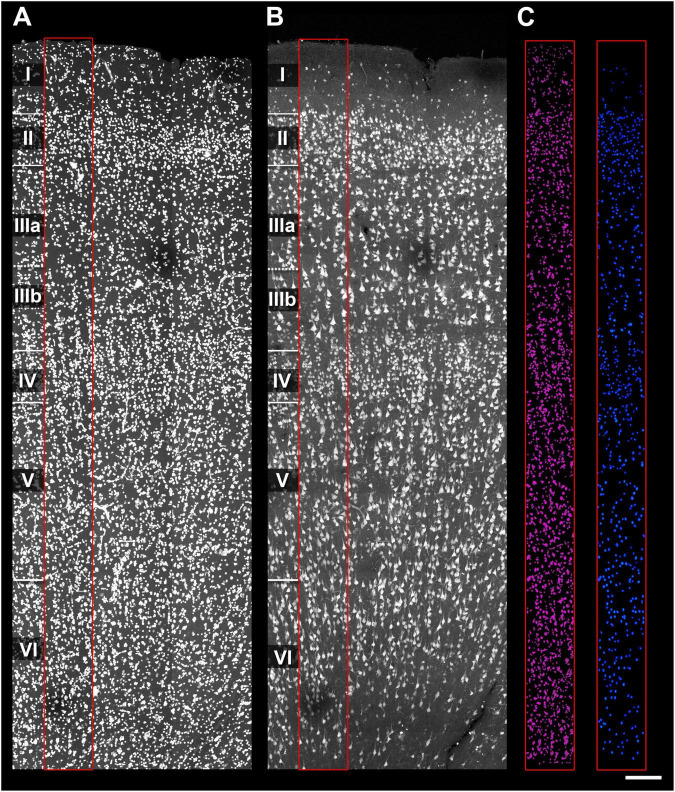
Cell segmentation in human samples. **(A)** Human BA21 section labeled with DAPI. Cortical layers I–VI are indicated. Red rectangle indicates the X and Y dimension of the region of interest (ROI) used for cell segmentation. **(B)** Same human BA21 section immunolabeled with NeuN. The red rectangle indicates the same ROI as in panel **(A)**. **(C)** 3D segmentation and reconstruction of the cells labeled with DAPI (purple) and NeuN (blue). Notice that DAPI segmentation includes more cells than NeuN, as it contains non-neuronal cell bodies. Scale [in panel **C**)]: 160 μm in panels **(A–C)**.

FIJI software (ImageJ 1.51 h; NIH, United States) was used to split channels from each optical series. Colocalization analysis and semi-automatic 3D segmentation were performed using EspINA software (EspINA Interactive Neuron Analyzer;^1^ Morales et al., 2011). Segmentation was carried out by expert neuroanatomists for all sections as follows: fluorescently labeled cell nuclei were individually segmented and visualized across channels simultaneously; each 3D segmented nucleus was numbered and tagged according to its staining pattern, nuclei size and shape. EspINA’s multichannel visualization facilitated classification of segmented nuclei as neurons (NeuN-ir) or non-neuronal cells (Figure 2). Data were exported as spreadsheets containing characteristics for each segmented cell.

**FIGURE 3:**
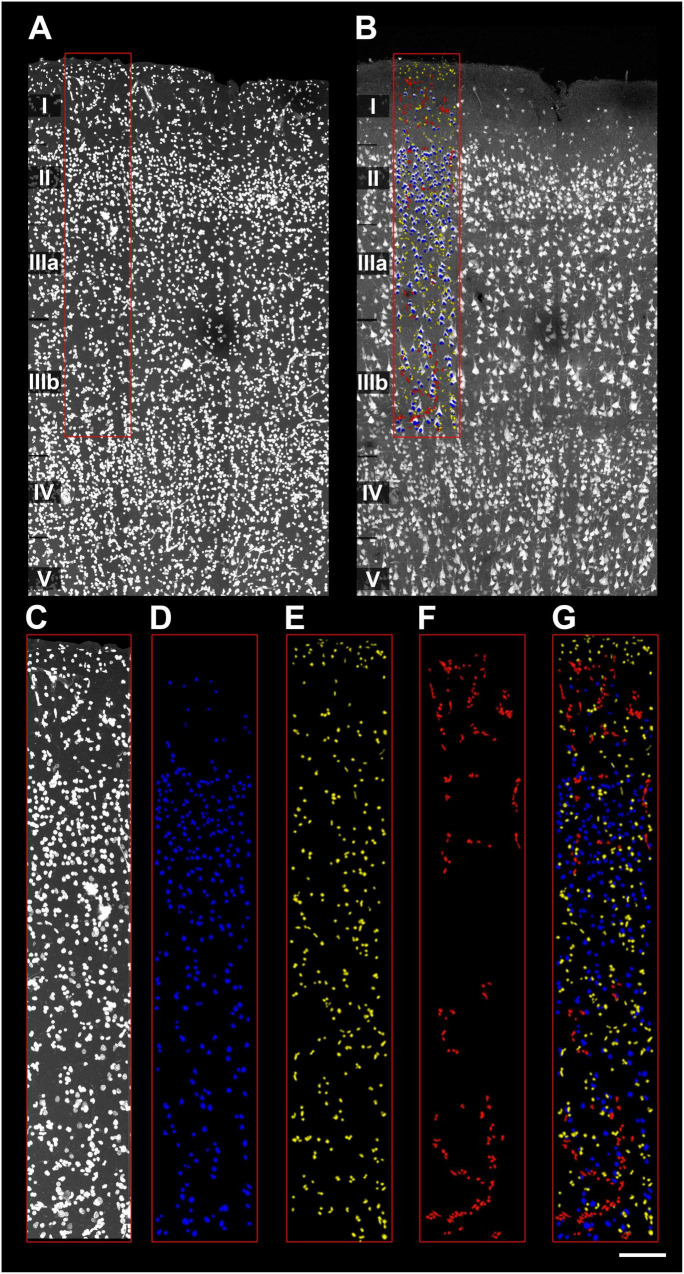
Detail of cell segmentation and data extraction from human samples. **(A)** Upper layers of a human BA21 section labeled with DAPI. Cortical layers I–V are indicated. Red rectangle indicates the X and Y dimension of the ROI used for cell segmentation. **(B)** Same human BA21 section immunolabeled with NeuN. The red rectangle indicates the same ROI as in panel **(A)**. 3D segmentation of both non-neuronal and neuronal cells (separated by colors) is overlaid over the ROI. **(C–G)** Example of the cell segmentation procedure. **(C)** ROI used for cell segmentation in upper layers (I–IV) labeled with DAPI. Neurons **(D)** were identified based on their presence in the NeuN channel, using EspINA software. Glial **(E)** and vascular cells **(F)** were identified in the DAPI channel. Vascular cells were classified based on their characteristic cylinder distribution and filiform shape (for a detailed description see Figure 4). **(G)** Superposition of the different cell segmentations. Scale [in panel **G**)]: 167 μm in panels **(A,B)**; 106 μm in panels **(C–G)**.

**FIGURE 4:**
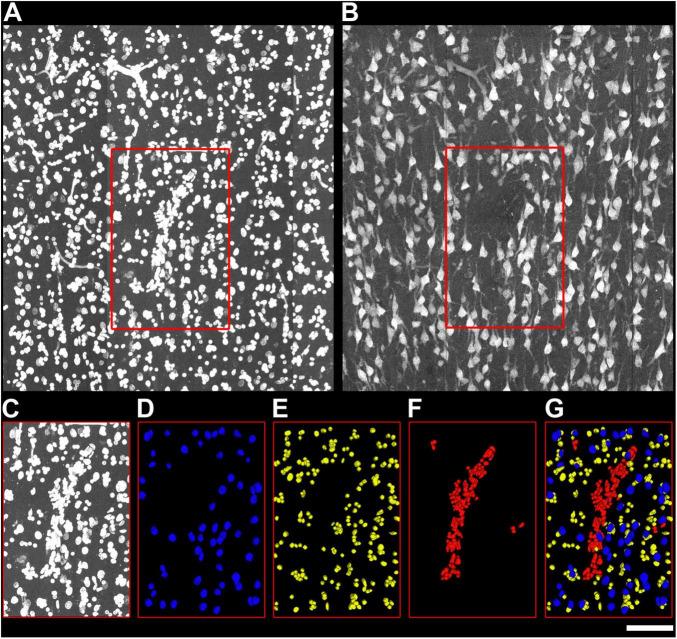
Example of vascular cell identification and segmentation in human samples. **(A)** Detail of a putative blood vessel in which vascular cells are labeled with DAPI, indicated in the red rectangle. Note the agglomeration of vascular cells, forming a characteristic cylinder distribution. **(B)** Same section labeled with NeuN. The red rectangle is placed in the same position as in panel **(A)**. Notice that the vessel structure is absent, indicating that cells identified in panel **(A)** as vascular cells are not neurons. **(C–G)** Example of segmentation of vascular cells. **(C)** Detail indicated as a red rectangle in panels **(A,B)**. Similar to Figure 3, neurons **(D)** were identified based on their presence in the NeuN channel, while glial **(E)** and vascular cells **(F)** were identified in the DAPI channel. Note the distinct distribution of vascular cells **(F)**. See Supplementary Video 1 for a visualization of the vascular cells detailed in the figure through the *Z*-axis. **(G)** Superposition of the different cell segmentations. In this example, a total of 402 cells were segmented and 3D reconstructed, in a volume of 0.003 mm^3^. Of these, 62 were identified as neurons, 225 as glial cells and 115 as vascular cells. Scale [in panel **G**)]: 87 μm in panels **(A,B)**; 81 μm in panels **(C–G)**.

In human BA21 sections, all DAPI+ nuclei were segmented in 200-μm-wide ROIs spanning the full cortical depth (layers I–VI) from two 50-μm-thick sections per subject (AB2, AB3, AB7; Figure 3). NeuN-ir nuclei were tagged as neurons, while vascular cells (DAPI+ nuclei with characteristic cylindrical/filiform distribution) were identified and tagged to distinguish them from glia (Figure 4 and Supplementary Video 1).

**FIGURE 5:**
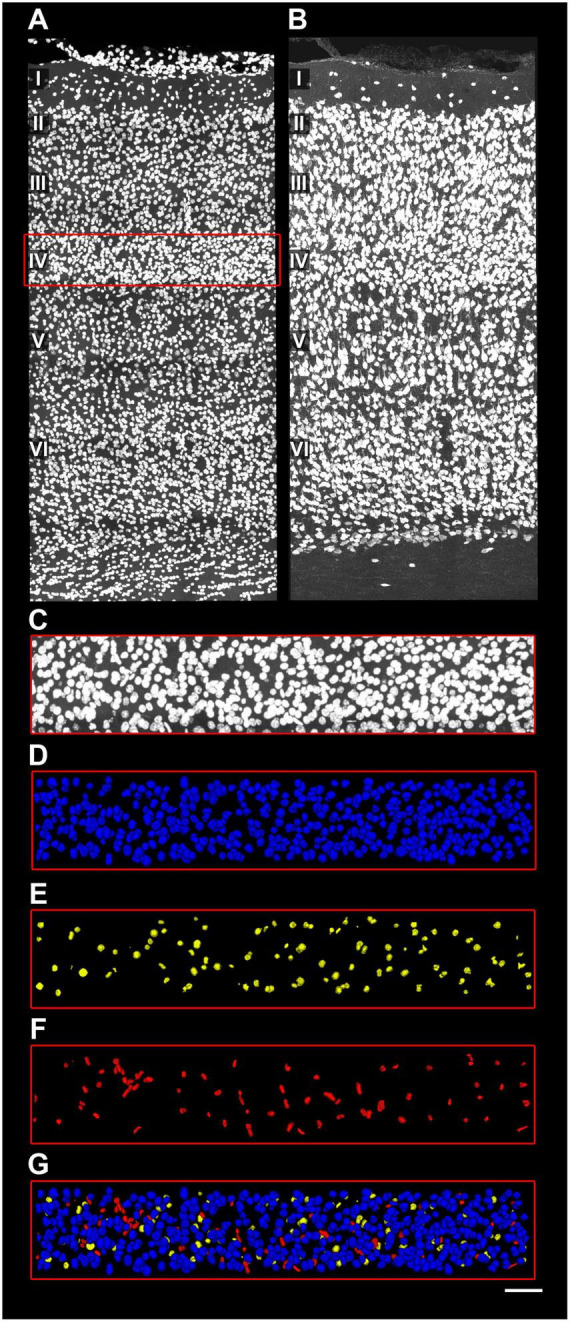
Detail of cell segmentation and data extraction from mice samples. **(A)** Mice S1HL section labeled with DAPI. Cortical layers I–VI are indicated. Red rectangle indicates the X and Y dimension of the ROI used for cell segmentation in layer IV. **(B)** Same S1HL section immunolabeled with NeuN. **(C–G)** Example of the cell segmentation procedure in mice samples. **(C)** ROI used for cell segmentation in layer IV, labeled with DAPI. Neurons **(D)** were identified based on their presence in the NeuN channel, using EspINA software. Glial **(E)** and vascular **(F)** cells were identified in the DAPI channel. Vascular cells were classified based on their characteristic cylinder distribution and colocalization with RECA immunolabeling. **(G)** Superposition of the different cell segmentations. Scale [in panel **G**)]: 90 μm in panels **(A,B)**; 45 μm in panels **(C–G)**.

In mouse S1HL sections, all DAPI+ nuclei were segmented in 100–600-μm-wide ROIs spanning layers I–VI from 50-μm-thick sections. NeuN-ir nuclei were tagged as neurons. In immersion-fixed samples, RECA-1 labeling specifically stained blood vessels, facilitating the distinction and tagging of vascular cells separate from other non-neuronal populations (Figure 5).

In both human and mouse samples, each ROI is formed by a rectangular prism enclosed by three acceptance planes and three exclusion planes marking its boundaries. All cells within the ROI were counted, as were those intersecting any of the acceptance planes. Cells that were outside the ROI, or intersecting any of the exclusion planes, were not counted.

### 2.5 Digital illustrations

Z-stack projection images were generated using FIJI software (ImageJ 1.51 h; NIH, United States). Snapshots of 3D visualizations and representations of segmented nuclei were created with EspINA software. Figures were assembled digitally using Photoshop CS4 (Adobe Inc., San Jose, CA, United States), with contrast, brightness, and sharpness adjusted as needed for optimal presentation.

### 2.6 Statistical analyses

Comparisons between fixation protocols (perfusion vs. immersion) and cortical layers in both human and mouse samples were performed using a generalized linear mixed model (GLMM), followed by a Turkey *post-hoc* test for multiple comparisons. All statistical analyses were conducted using GraphPad Prism (version 9 for Windows; GraphPad Software Inc., United States) and R (version 4.5.1; Bell Laboratories, NJ, United States).^2^

## 3 Results

### 3.1 Cell numbers and types in S1HL from mouse brain

3D segmentation and counting were performed in coronal sections containing the S1HL cortical region, obtained from cortical samples fixed either by perfusion or by immersion. Samples fixed by immersion were divided into two subgroups: (1) brains fixed immediately after sacrifice, and (2) brains fixed after a 5-h PMT (to mimic the postmortem interval of human autopsy samples). Segmentation was performed on an estimated total cortical tissue volume of ∼0.075 mm^3^, corresponding to the cumulative volume of all analyzed sections.

The 3D segmented cells from all animals and layers totaled 25,003 DAPI-labeled cell nuclei. Of these, 15,567 (∼62%) were identified as neurons (confirmed by NeuN-ir), and 9,436 as non-neurons (lacking NeuN-ir). In the immersion-fixed samples, 2,168 of the non-neuronal cells were identified as vascular cells based on their characteristic cylindrical distribution and colocalization with RECA-1 labeling (Figure 5). Mouse cortical samples displayed a similar proportion of neurons across both fixation protocols (63% for immersion and 61% for perfusion; Table 2). This proportion was also similar between the two immersion subgroups, indicating that PMT did not affect cell counting (Table 2).

**TABLE 2:** Proportion of cells identified as neurons (Neun-ir) in the mouse S1HL (fixed by immersion and perfusion).

Layer	Perfusion (mean ± SD)	Immersion PMT 0 h (mean ± SD)	Immersion PMT 5 h (mean ± SD)
I	0.20 ± 0.02 (20%)	0.14 ± 0.00 (14%)	0.14 ± 0.05 (14%)
II	0.70 ± 0.03 (70%)	0.69 ± 0.05 (69%)	0.73 ± 0.01 (73%)
III	0.64 ± 0.07 (64%)	0.69 ± 0.03 (69%)	0.66 ± 0.01 (66%)
IV	0.71 ± 0.06 (71%)	0.71 ± 0.02 (71%)	0.74 ± 0.01 (74%)
V	0.50 ± 0.02 (50%)	0.53 ± 0.00 (53%)	0.52 ± 0.01 (52%)
VI	0.69 ± 0.02 (69%)	0.66 ± 0.01 (66%)	0.70 ± 0.02 (70%)
I–VI	0.61 ± 0.05 (61%)	0.63 ± 0.02 (63%)	0.63 ± 0.01 (63%)

Values in parentheses refer to the percentage of cells identified. Proportion for non-neuronal cells are detailed in Supplementary Tables 1, 3, 5. Values for all layers (I–VI) correspond to the accumulated value. Supplementary Tables 2, 4,6 include percentages per individual animal. PMT, Postmortem time between death and fixation; SD, Standard Deviation.

**FIGURE 6:**
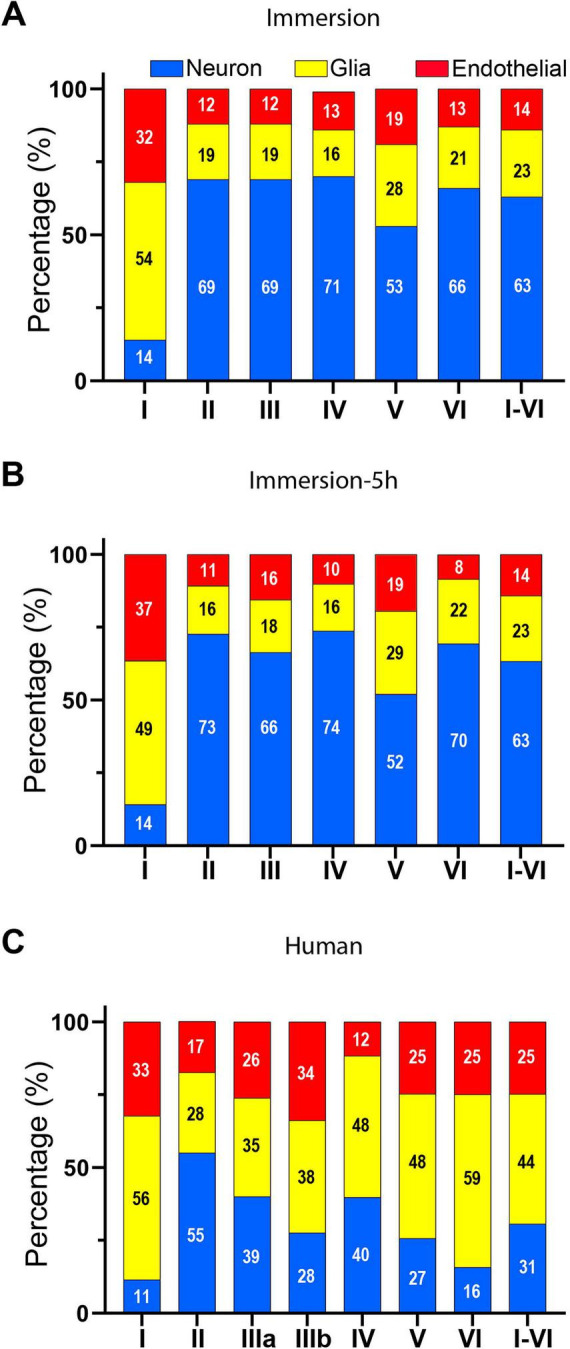
Analysis of non-neuronal and neuronal proportions in mice and human samples, separated by cortical layer. **(A,B)** Bar plot of the percentages of neurons (blue) glia (yellow) and vascular cells (red) in mice samples fixated by immersion. Values are indicated in the graphs and in Table 2 and Supplementary Tables 1–4. Regardless of the fixative protocol used, layer I and layer V had a significantly lower proportion of neurons than the rest of the layers (GLMM, *p* < 0.0001). **(C)** Bar plot of the percentages of non-neuronal and neuronal cells in human samples. Values are indicated in the graphs and in Table 4 and Supplementary Table 13. Layer I exhibited the lowest proportion of neurons compared to the rest of the layers, except for layer VI (GLMM, *p* < 0.0001).

Since cell segmentation covered the full extent of the S1HL cortical column (from pia to white matter), further analyses were conducted for each cortical layer, allowing for separate final estimates (Table 2). Neurons predominated over non-neuronal cells in all layers except layer I (Figure 6 and Table 2). However, significant differences in cell proportions were observed between layers in both perfusion- and immersion-fixed samples. Specifically, layer I presented the lowest proportion of neurons (20 and 14% in perfusion and immersion groups, respectively), differing significantly from all other layers (GLMM, *p* < 0.0001; Table 2). Layer V also showed a significantly lower proportion of neurons compared to other layers (GLMM, *p* < 0.0001; Table 2). Layers II, III, IV, and VI had relatively similar neuron percentages in both fixative groups (Table 2), although GLMM analysis revealed some significant differences in the proportion of neurons between these layers, in either the perfusion or immersion fixative group (specific *p*-values for every layer comparison are detailed in Supplementary Tables 7–9).

### 3.2 Proportion of neurons and layer differences

To estimate the approximate number of non-neuronal cells per neuron, a ratio was calculated by dividing the number of non-neurons (NeuN-negative, thus including both glia and vascular cells) by the number of neurons (NeuN-positive) in all layers for both fixation protocols (Table 3). When considering cumulative values from all cortical layers, this ratio was very similar for the two sample types (Table 3). Furthermore, both fixation protocols displayed similar ratios in all cortical layers except layer I, which contained a much larger number of non-neuronal cells per neuron in both sample types. Layers II through VI presented values less than or equal to 1, indicating one or fewer non-neuronal cells per neuron, with no differences found between fixation protocols. However, in layer I, the number of non-neuronal cells per neuron in immersion-fixed samples was 6.3, which was higher than the ratio estimated in perfusion-fixed samples (4.1; Table 3).

**TABLE 3:** Estimated ratio of non-neurons per neuron and glia per neuron in each cortical layer in S1HL mouse samples, per fixation protocol.

	Perfusion	Immersion PMT 0 h		Immersion PMT 5 h	
Layer	Non-neuron/neuron ratio (mean ± SD)	Non-neuron/neuron ratio (mean ± SD)	Glia/neuron ratio (mean ± SD)	Non-neuron/neuron ratio (mean ± SD)	Glia/neuron ratio (mean ± SD)
I	4.1 ± 0.39	6.1 ± 0.06	3.9 ± 0.01	6.5 ± 2.38	3.8 ± 1.50
II	0.4 ± 0.06	0.5 ± 0.01	0.3 ± 0.07	0.4 ± 0.01	0.2 ± 0.00
III	0.6 ± 0.17	0.5 ± 0.06	0.3 ± 0.04	0.5 ± 0.02	0.3 ± 0.01
IV	0.4 ± 0.11	0.4 ± 0.03	0.2 ± 0.04	0.4 ± 0.02	0.2 ± 0.02
V	1.0 ± 0.06	0.9 ± 0.01	0.5 ± 0.00	0.9 ± 0.03	0.6 ± 0.05
VI	0.4 ± 0.04	0.5 ± 0.02	0.3 ± 0.08	0.4 ± 0.04	0.3 ± 0.04
I–VI	0.7 ± 0.15	0.6 ± 0.05	0.4 ± 0.00	0.6 ± 0.02	0.4 ± 0.01

Values for all layers (I–VI) correspond to the accumulated value. Supplementary Tables 10–12 include percentages per individual animal. PMT, Postmortem time between death and fixation; SD, Standard Deviation.

### 3.3 Non-neuronal cells per neuron and glia-to-neuron ratio

Finally, in the immersion-fixed groups, since we were able to differentiate vascular cells from other non-neuronal cells (presumably glia), we could exclude vascular cells to obtain a true glia-to-neuron ratio (GNR). In both immersion subgroups, the overall GNR was 0.4, again confirming that PMT did not affect cell number or distribution. As with the non-neuron-to-neuron ratio, layer I presented the highest GNR (3.8), whereas the remaining layers displayed consistently lower values (ranging from 0.2 in layers II and IV to 0.6 in layer V; Table 3).

### 3.4 Cell numbers and types in the human temporal cortex

3D segmentation and counting of labeled cells were performed in sections obtained from three human brain autopsy samples. As described for mouse brain tissue, the analysis was conducted by cortical layer, allowing for separate final estimates for each layer (Table 4). Layers I, II, IIIa, IIIb, IV, V, and VI were identified in Brodmann area 21. Segmentation was performed on an estimated total cortical tissue volume of 0.39 mm^3^, corresponding to the cumulative volume of all analyzed sections.

**TABLE 4:** Proportion of cells identified as neurons, glia and vascular cells in the human BA21.

Layer	Neuron (mean ± SD)	Glia (mean ± SD)	Vascular (mean ± SD)
I	0.11 ± 0.02 (11%)	0.56 ± 0.05 (56%)	0.33 ± 0.07 (33%)
II	0.55 ± 0.01 (55%)	0.28 ± 0.02 (28%)	0.17 ± 0.02 (17%)
IIIa	0.39 ± 0.04 (39%)	0.35 ± 0.05 (35%)	0.26 ± 0.04 (26%)
IIIb	0.28 ± 0.03 (28%)	0.38 ± 0.04 (38%)	0.34 ± 0.03 (34%)
IV	0.40 ± 0.03 (40%)	0.48 ± 0.12 (48%)	0.12 ± 0.09 (12%)
V	0.27 ± 0.04 (27%)	0.48 ± 0.07 (48%)	0.25 ± 0.04 (25%)
VI	0.16 ± 0.01 (16%)	0.59 ± 0.04 (59%)	0.25 ± 0.03 (25%)
I–VI	0.31 ± 0.02 (31%)	0.44 ± 0.06 (44%)	0.25 ± 0.03 (25%)

Values in parentheses refer to the percentage of cells identified. Values for all layers (I–VI) correspond to the accumulated value. Supplementary Table 13 includes percentages per individual case. SD, Standard Deviation.

The 3D segmented cells from all individuals and layers totaled 13,473 DAPI-labeled cell nuclei. Of these, 4,135 were identified as neurons (confirmed by NeuN-ir) and 9,338 as non-neurons (lacking NeuN-ir). Among the non-neuronal population, 3,336 cells were identified as vascular cells. The remaining 6,002 non-neuronal cells were considered glial cells. Based on these counts, human BA21 samples displayed an average composition of 31% neurons and 69% non-neurons (44% glia plus 25% vascular cells; Figure 6 and Table 4).

### 3.5 Proportion of neurons and layer differences

The proportion of neurons were calculated separately for each cortical layer, with contingency tables and multiple comparisons revealing significant differences between layers. For instance, layer II displayed the highest proportion of neurons (55%; Table 4), differing significantly from all other layers (GLMM, *p* < 0.0001; Figure 6 and Table 4). Conversely, layer I showed the lowest proportion (11%), which was also significantly lower than all other layers (GLMM, *p* < 0.0001; Figure 6 and Table 4), except for layer VI. Layers IIIa and IV showed no significant difference, displaying similar neuronal proportions (39 and 40%, respectively), as did layers IIIb and V (28 and 27%, respectively; Figure 6 and Table 4). Some additional differences in the proportion of neurons between layers were revealed by the GLMM analysis, as detailed in Supplementary Table 14.

### 3.6 Non-neuronal cells per neuron and glia-to-neuron ratio

A ratio estimating the approximate number of non-neuronal cells per single neuron was calculated for all layers, following the same methodology as that used for mouse samples (Table 5). Based on cumulative data from all layers, the number of non-neuronal cells per neuron in human BA21 was 2.3, indicating more than two non-neuronal cells for every neuron. Layer I displayed the highest number of non-neuronal cells per neuron (∼8), followed by layer VI (∼5), whereas the remaining layers showed values ranging from 0.8 (layer II) to approximately 3 (layer V; Table 5).

**TABLE 5:** Estimated ratio of non-neurons per neuron and Glia per neuron in each cortical layer of human BA21.

Layer	Non-neuron/neuron ratio (mean ± SD)	Glia/neuron ratio (mean ± SD)
I	7.9 ± 1.40	4.9 ± 0.43
II	0.8 ± 0.02	0.5 ± 0.02
IIIa	1.6 ± 0.25	0.9 ± 0.19
IIIb	2.6 ± 0.34	1.4 ± 0.27
IV	1.5 ± 0.19	1.2 ± 0.39
V	2.8 ± 0.54	1.8 ± 0.55
VI	5.3 ± 0.58	3.8 ± 0.60
I–VI	2.3 ± 0.26	1.5 ± 0.30

Values for all layers (I–VI) correspond to the accumulated value. Supplementary Table 15 includes values per individual case. SD, Standard Deviation.

Furthermore, the glia-to-neuron ratio (GNR) was calculated. Across all layers, the overall GNR was 1.5, although this value varied significantly between layers. Layer I presented the highest GNR (∼5), whereas layer II had the lowest ratio (∼0.5; Table 5).

### 3.7 Comparison of mouse and human samples

**FIGURE 7:**
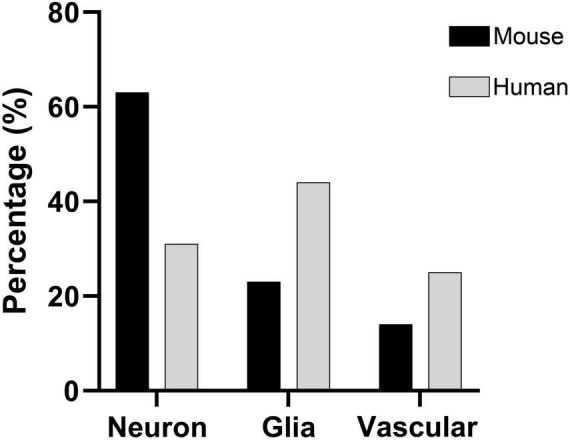
Comparison of cell percentages between mouse and human samples. The mouse S1HL region (black column) contains a significantly higher percentage of neurons than human BA21 (gray column; GLMM, *p* < 0.0001). Mouse data were extracted from the immersion fixative group (see Table 2).

Comparison of mouse and human samples indicated significant differences in the proportions of neurons and non-neuronal cells. The overall proportion of neurons in mouse samples was significantly larger than in human samples (GLMM, *p* < 0.0001; Figure 7). Moreover, the ratio of non-neuronal cells per neuron in the mouse was smaller than in the human brain (0.6 in mouse samples vs. 2.3 in human sections).

## 4 Discussion

In the present study, we quantified the proportion of neurons, glia, and vascular cells (primarily endothelial cells) of the mouse and human cerebral cortex using a direct counting method. In this paper, the term cortical column refers to a piece of cortical tissue of arbitrary width extending from the pial surface to the gray–white matter interface. Within these columns, we also obtained detailed laminar estimates of the glia-to-neuron ratio (GNR) and the non-neuron-to-neuron ratio (nNNR), thereby enhancing current knowledge of cortical cellular organization. The present results reveal marked species-specific differences in these cellular proportions.

### 4.1 Differential laminar distribution of cortical cell types

Neurons located in distinct cortical layers establish specific connections locally and with other cortical and subcortical regions, providing a key anatomical basis for the columnar organization hypothesis of the cerebral cortex (e.g., Jones and Peters, 1987; Lund, 1988; White, 1989; Rockland and Ichinohe, 2004; DeFelipe et al., 2007, 2012). The number and types of neurons in these layers are critically related to the pattern of such connections and, consequently, to the overall structural organization of each cortical area (DeFelipe, 2015; Harris and Shepherd, 2015; Larkum et al., 2018).

From an anatomical perspective, the quantitative distribution of neurons across layers is a key determinant of the laminar patterns of afferent and efferent connectivity that define regional specificity within the cortex. Equally important is the proportion of non-neuronal cells—primarily glia and vascular cells—which varies across layers and contributes to the structural, metabolic, and homeostatic support required for laminar specialization (von Bartheld et al., 2016; Verkhratsky and Nedergaard, 2016). Given that cortical layers function collectively within vertically organized cortical columns, the canonical microcircuit of each column depends on the balance among neuronal density, glial populations, and local microvasculature. Quantifying the relative proportions of neurons, glia, and vascular cells is therefore essential to understand how columnar architecture supports information processing and to identify region-specific features of cortical organization (DeFelipe, 2015; Harris and Shepherd, 2015; Larkum et al., 2018).

### 4.2 Limitations of traditional counting methods

Three main classes of methods have historically been used for cell quantification in the brain (von Bartheld et al., 2016). Traditional histological approaches (e.g., Haug et al., 1984; Haug, 1987; Pakkenberg and Gundersen, 1988; Schmitz and Hof, 2005; Lyck et al., 2009) were followed by stereological methods, which remain widely applied. Stereology has been instrumental for estimating cell populations, but it faces several challenges, particularly with regard to (i) ensuring that the chosen sample is representative of large, heterogeneous structures and (ii) avoiding double counting of elements in serial sections (von Bartheld, 2001, 2002; Schmitz and Hof, 2005). These limitations make stereological approaches technically demanding and sensitive to bias if parameters such as section number, slice spacing, and region-of-interest definition are not carefully optimized.

An alternative, the isotropic fractionator (IF), yields estimates that are equivalent in precision to stereological approaches (Herculano-Houzel et al., 2015) and allows rapid quantification of large numbers of cells. However, the homogenization process and mechanical disruption of brain tissue may compromise nuclear membrane integrity and lead to underestimation of total cell counts (Carlo and Stevens, 2013; Charvet et al., 2015). Furthermore, since this approach relies solely on the counting of labeled nuclei in homogenized tissue (e.g., NeuN or DAPI), cell classification depends entirely on staining specificity. Certain populations, such as vascular nuclei, may therefore be underestimated if they are not effectively labeled. Importantly, the IF does not preserve spatial or laminar organization of neuronal and non-neuronal populations, which limits its utility for studies that require precise anatomical localization.

### 4.3 Advantages of 3D direct quantification

The methodological approach used in the present study, although time-consuming, overcomes several of these limitations. It enables direct quantification of cells within a defined volume of tissue on the basis of specific immunolabeling, cell morphology, and spatial distribution across cortical layers. This makes it possible to directly calculate the number and proportion of different cell types (neurons, glia, and vascular cells) and to compare brain regions and species using the same methodology. In this study, more than 25,000 cells in the mouse cortex and 13,000 cells in the human cortex were identified and classified according to cell type. Additionally, because cell segmentation is performed in a 3D environment, this approach enables the exploration of spatial aspects of the volumetric distribution of the different cell types that constitute a cortical column. For this purpose, we utilized the EspINA software tool, which was developed in our laboratory (Morales et al., 2011) for both quantitative analysis and the examination of spatial characteristics of microanatomical elements. This includes the identification of neuronal types and the analysis of synapses across the entire cortical thickness in the cerebral cortex (e.g., Montero-Crespo et al., 2020; Tapia-González and DeFelipe, 2023; Cano-Astorga et al., 2023, 2024; Plaza-Alonso et al., 2025).

### 4.4 Species-specific differences in cellular ratios

**FIGURE 8:**
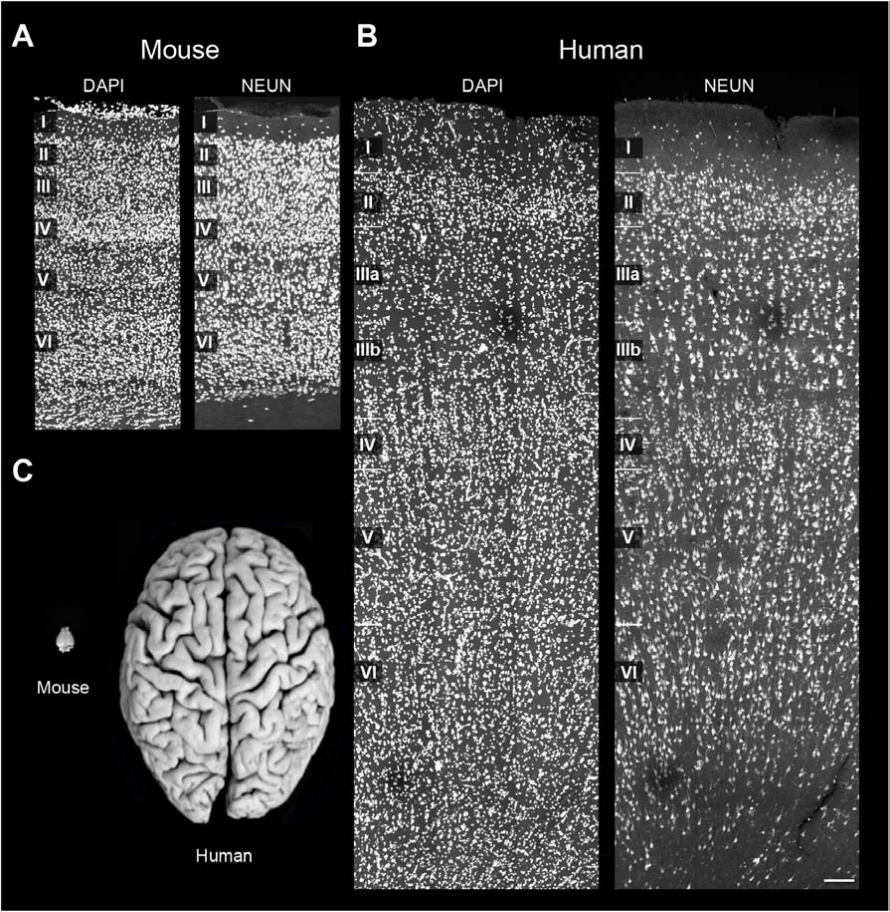
Comparison of the cellular composition from the mouse and human cerebral cortex. **(A)** Mice S1HL section labeled with DAPI (left) and NeuN (right). Cortical layers I–VI are indicated. **(B)** Human BA21 section labeled with DAPI (left) and NeuN (right). Cortical layers I–VI are indicated. Both mouse and human sections were taken at the same magnification. Note the remarkable differences regarding cortical thickness and cellular density between mouse and human sections across the full extent of the cortical column. **(C)** Photograph of a mouse (left) and human (right) brain, taken at the same magnification. Modified from DeFelipe (2011). Scale [in panel **B**)]: 130 μm in panels **(A,B)**; 1.76 cm in panel **(C)**.

The present study revealed marked differences in cellular proportions between the mouse and human cortex. Although the analysis compared distinct regions with different functions—the S1HL in the mouse frontal cortex and BA21 in the human temporal cortex—both are considered isocortical areas. Notably, significant species-specific variations were observed: neurons represented approximately 60% of all cells in the mouse S1HL, whereas this proportion was lower—around 30%—in the human temporal cortex. This difference was clearly reflected in the non-neuron-to-neuron ratio (nNNR) and the glia-to-neuron ratio (GNR). In all layers of the mouse S1HL, both ratios were below one (0.6 for nNNR and 0.4 for GNR), whereas in the human samples, the nNNR reached 2.3 and the GNR was 1.5 when considering all layers combined. Figure 8 illustrates sections of the cerebral cortex of the mouse and human at the same magnification, labeled for DAPI and NeuN. These images clearly demonstrate not only the significant differences in cortical thickness between the mouse and human, but also the variation in cell densities across the full extent of the cerebral cortex, from the pial surface to the white matter interface. Furthermore, the figure highlights that these cellular differences do not scale linearly with brain size, especially considering that the human brain is approximately 3,000 times larger than the mouse brain by volume.

Both the nNNR and the GNR are regarded as key descriptors of brain structure and neuronal function, given the critical role of glial cells in supporting neuronal physiology and metabolism (Barres, 2008; Lee et al., 2012; Schummers et al., 2008; Ullian et al., 2001; Verkhratsky and Nedergaard, 2016). Historically, the concept of a 10:1 glia-to-neuron ratio in the human brain predominated in neuroscience and was incorporated into major textbooks (e.g., Kandel et al., 1995, 2000). This notion, originally proposed by Hyden (1960), lacks experimental support and, in light of the present results, clearly overestimates glial abundance in the cerebral cortex. Instead, our data are generally consistent with numerous studies using histological, stereological, and IF approaches (Friede, 1954; Hawkins and Olszewski, 1957; Pelvig et al., 2003, 2008; Azevedo et al., 2009; Ribeiro et al., 2013; von Bartheld et al., 2016; see Table 6). Although reported literature values are variable and often lack laminar-specific information, most studies describe GNR values ranging between approximately 1 and 2, aligning with the ratios obtained here. Notably, Ribeiro et al. (2013) reported an nNNR of 2.6 in the human temporal cortex using the IF, which is close to the 2.3 value observed in our analysis of human BA21.

**TABLE 6:** Previous reports on GNR and nNNR of several human cortical regions, analyzed with different methodological approaches.

GNR	nNNR	Brain region	Layers	Cases	Age	PMT	Method	References
2.72	-	DL_PFC	I–VI	11 Cases (8 male; 3 female)	4–15 yr	5–24 h	Stereology	Rabelo et al., 2023
1.63	-	BA32	II/III	6 Cases	< 55	Not specified	Stereology	Sherwood et al., 2006
2.19	-	BA4	II/III	6 Cases	< 55	Not specified	Stereology	Sherwood et al., 2006
1.55	-	BA44	II/III	6 Cases	< 55	Not specified	Stereology	Sherwood et al., 2006
-	2.3	BA9	II–VI	6 Cases	24–55	Not specified	DNA extraction	Hess and Thalheimer, 1971
1.65	-	BA9	II/III	6 Cases	< 55	Not specified	Stereology	Sherwood et al., 2006
-	2.71	Frontal	I–VI	1 Female	65	Perfusion	IF	Ribeiro et al., 2013
1.17	-	Frontal	Not specified	6 Female	60–80	4–48 h	Stereology	Karlsen and Pakkenberg, 2011
1.45	-	Frontal	I–VI	20 Cases (6 male; 14 female)	60–98	Not specified	Stereology	Pelvig et al., 2003
-	1.79	Frontal Lobe	I–VI	5 Female	71–84	< 24 h	IF	Andrade-Moraes et al., 2013
0.54	-	Left BA9	II–VI	11 Cases	47–80	Not specified	Histology	Diamond et al., 1985
0.57	-	Right BA9	II–VI	11 Cases	47–80	Not specified	Histology	Diamond et al., 1985
-	2.29	Prefrontal	I–VI	1 Female	65	Perfusion	IF	Ribeiro et al., 2013
-	5.43	Hippocampus + Amigdala	All layers	5 Female	71–84	< 24 h	IF	Andrade-Moraes et al., 2013
-	1.4	MTG	I–VI	2 Male	32; 36	Biopsy	MERFISH	Fang et al., 2022
-	1.5	STG	I-VI	2 Male	29; 42	Not specified	MERFISH	Fang et al., 2022
-	2.64	Temporal	I–VI	1 Female	65	Perfusion	IF	Ribeiro et al., 2013
1.05	-	Temporal	Not specified	6 Female	60–80	4–48 h	Stereology	Karlsen and Pakkenberg, 2011
1.42	-	Temporal	I–VI	20 Cases (6 male; 14 female)	60–98	Not specified	Stereology	Pelvig et al., 2003
1.78	-	Temporal cortex	II–VI	1 Female	36	Not specified	Histology	Hawkins and Olszewski, 1957
1.5	2.3	Temporal cortex	I–VI	3 Cases (1 female; 2 male)	53–66	2–4 h	Direct counting	Present study
-	2.21	Parietal	I–VI	1 Female	65	Perfusion	IF	Ribeiro et al., 2013
1	-	Parietal	Not specified	6 Female	60–80	4–48 h	Stereology	Karlsen and Pakkenberg, 2011
1.26	-	Parietal	I–VI	20 Cases (6 male; 14 female)	60–98	Not specified	Stereology	Pelvig et al., 2003
0.52	-	Left BA39	II–VI	11 Cases	47–80	Not specified	Histology	Diamond et al., 1985
0.49	-	Right BA39	II–VI	11 cases	47–80	Not specified	Histology	Diamond et al., 1985
0.68	-	BA17	II–VI	11 Cases (4 male; 7 female)	17–93	6–24 h	Stereology	Leuba and Garey, 1989
-	1.31	V1 (BA17)	I–VI	1 Female	65	Perfusion	IF	Ribeiro et al., 2013
0.76	1.1	BA17	I–VI	7 Male	23–66	6–27 h	Direct counting	Garcia-Marin et al., 2024
1.02	-	BA18	II–VI	11 Cases (4 male; 7 female)	17–93	6–24 h	Stereology	Leuba and Garey, 1989
0.8	-	Occipital	Not specified	6 Female	60–80	4–48 h	Stereology	Karlsen and Pakkenberg, 2011
1.31	-	Occipital	I–VI	20 Cases (6 male; 14 female)	60–98	Not specified	Stereology	Pelvig et al., 2003
-	1.71	Posterior	I–VI	1 Female	65	Perfusion	IF	Ribeiro et al., 2013
-	4.76	BA17 (striate cortex GM+WM)	I–WM	2 Cases (1 male; 1 female)	16–77	12–17 h	DNA extraction	Nurnberger and Gordon, 1957
-	2.10	Insula	I–VI	1 Female	65	Perfusion	IF	Ribeiro et al., 2013
-	1.56	Other lobes	I–VI	5 Female	71–84	< 24 h	IF	Andrade-Moraes et al., 2013
1.57–2.02	-	Total neocortex	Not specified	94 cases (62 male; 32 female)	18–93	Not specified	Stereology	Pakkenberg et al., 2003
1.32	-	Total neocortex	Not specified	18 Female	18–93	7–96 h	Stereology	Pelvig et al., 2008
1.49	-	Total neocortex	Not specified	13 Male	19–87	7–96 h	Stereology	Pelvig et al., 2008
-	1.48	Total neocortex	I–VI	4 Male	40–71	< 24 h	IF	Azevedo et al., 2009
1.24–1.98 (Av: 1.68)	-	Neocortex (unknown areas)	II–VI	Not specified	24–54	Not specified	Histology	Friede, 1954

Av, Average; BA, Brodmann Area; DL_PFC, Dorsolateral Prefrontal Cortex; GM, Gray Matter; IF, Isotropic Fractionator; MTG, Middle Temporal Gyrus; STG, Superior Temporal Gyrus; WM, White Matter.

### 4.5 Distinguishing glial and vascular cell populations

A critical distinction between the nNNR and GNR is the contribution of vascular cells, which was explicitly accounted for in the present study. While the nNNR includes both vascular and glial cells, the GNR exclusively represents the glial population. Notably, studies unable to differentiate between these two non-neuronal types—particularly those utilizing the IF technique—often assume that the nNNR provides a reliable approximation of the true GNR. However, histological studies indicate that approximately 30% of all non-neuronal cells in the neocortex are vascular cells (Brasileiro-Filho et al., 1989; Bjugn and Gundersen, 1993; Lyck et al., 2009; García-Amado and Prensa, 2012; Bahney and von Bartheld, 2018; von Bartheld, 2018). Consistent with these reports, our data show that vascular cells constitute 36% of all non-neuronal cells in the human BA21 across all layers. Consequently, the nNNR should be regarded as a maximum estimate for the actual glia-to-neuron ratio (von Bartheld, 2018).

### 4.6 Evolutionary and metabolic considerations

The nNNR (0.6) and GNR (0.4) values obtained for the mouse cortex in the present analysis are consistent with previous reports, which generally describe ratios below 1.0, indicating that there is < 1 non-neuronal (or glial) cell per neuron in the mouse brain (Friede, 1954; Herculano-Houzel et al., 2006; Herculano-Houzel, 2014). From a comparative perspective, variation in these ratios across species provides valuable insights into brain organization. While it was traditionally proposed that the GNR scales positively with brain size (Hawkins and Olszewski, 1957), more recent evidence suggests that the proportion of glial cells correlates with both neuronal density and neuronal size. Consequently, brain regions with lower neuronal densities often contain larger numbers of glial cells and a higher GNR, which has been hypothesized to reflect the increased metabolic demands of larger neurons (Hawkins and Olszewski, 1957; Attwell and Laughlin, 2001; Karbowski, 2007; Quintela-López et al., 2022; Herculano-Houzel, 2014). By contrast, Herculano-Houzel (2011) estimated that average glucose consumption per neuron remains remarkably constant across species and that variations in neuronal density or GNR do not correlate directly with metabolic rate (Herculano-Houzel, 2011, 2014). Instead, it has been proposed that differences in GNR across brain regions and species arise from mechanical limitations to glial proliferation during development, rather than reflecting large variations in neuronal energy requirements (Herculano-Houzel, 2014).

## 5 Conclusion

The present study provides an accurate direct quantification of cellular proportions in the mouse and human cerebral cortex, based on the manual identification of tens of thousands of cells while preserving laminar and spatial context. By precisely estimating the GNR and nNNR, this approach overcomes several limitations associated with previous counting methods and allows for the validation of data obtained using different techniques. Future extensions could incorporate subtype-specific markers to determine pyramidal:interneuron and astrocyte:oligodendrocyte:microglia ratios, potentially revealing laminar-specific scaling rules. The archived 3D datasets (>38,000 segmented cells) provide an ideal foundation for such analyses.

## Data Availability

The original contributions presented in the study are included in the article/Supplementary material, further inquiries can be directed to the corresponding author.

## Footnotes

1
https://cajalbbp.csic.es/en/espina-2/

2
http://www.R-project.org
