# Supplementary material for: Layer-specific cellular composition of mouse primary somatosensory and human temporal cortex: a direct 3D confocal counting approach
Source: Front Neuroanat. 2026 Apr 23;20:1795807. doi: 10.3389/fnana.2026.1795807 (PMC13150353; doi:10.3389/fnana.2026.1795807)
Supplement: Supplementary file 2 [file Data_Sheet_1.doc]

**Supplementary Material:**

Layer-specific cellular composition of mouse primary somatosensory and human temporal cortex: a direct 3D confocal counting approach

Sergio Plaza-Alonso^1,2^, Lidia Alonso-Nanclares^1,2^, Silvia Tapia-Gonzalez^1,3^, Laura Fernández-García^1^, Asta Kastanauskaite^1^, Javier DeFelipe^1,2,^*

1. Laboratorio Cajal de Circuitos Corticales (CTB), Universidad Politécnica de Madrid, Campus de Montegancedo s/n, Pozuelo de Alarcón 28223, Madrid, Spain

2. Cajal Neuroscience Center, CSIC, Avenida de León 1, Alcalá de Henares 28805, Madrid, Spain

3. Departamento de Ciencias Médicas Básicas, Facultad de Medicina-Instituto de Medicina Molecular Aplicada-Nemesio Díez (IMMA-ND), Universidad San Pablo-CEU, CEU Universities, Urbanización Montepríncipe, Madrid, Spain

This document includes:

**Supplementary Tables 1–15**

Additional supplementary material includes **Video 1**

**Supplementary Table 1.** Proportion of cells identified as neurons, glia and vascular cells in the mouse S1HL, fixated by immersion with no post-mortem delay. Values in parentheses refer to the percentage of cells identified. Values for all layers (I–VI) correspond to the accumulated value. SD: Standard Deviation

| **Layer** | **Neuron (mean ± SD)** | **Glia (mean ± SD)** | **Vascular (mean ± SD)** |
| --- | --- | --- | --- |
| **I** | 0.14 ± 0.00 (14%) | 0.54 ± 0.00 (54%) | 0.32 ± 0.01 (32%) |
| **II** | 0.69 ± 0.05 (69%) | 0.19 ± 0.03 (19%) | 0.12 ± 0.07 (12%) |
| **III** | 0.69 ± 0.03 (69%) | 0.19 ± 0.02 (19%) | 0.12 ± 0.04 (12%) |
| **IV** | 0.71 ± 0.02 (71%) | 0.16 ± 0.02 (16%) | 0.13 ± 0.01 (13%) |
| **V** | 0.53 ± 0.00 (53%) | 0.28 ± 0.00 (28%) | 0.19 ± 0.00 (19%) |
| **VI** | 0.66 ± 0.01 (66%) | 0.21 ± 0.05 (21%) | 0.13 ± 0.06 (13%) |
| **I–VI** | 0.63 ± 0.02 (63%) | 0.23 ± 0.01 (23%) | 0.14 ± 0.03 (14%) |

|  |  | **Neuron (%)** | **Glia (%)** | **Vascular (%)** |
| --- | --- | --- | --- | --- |
| **I** | **4c** | 13.9 (33) | 54.0 (128) | 32.1 (76) |
|  | **6c** | 14.1 (22) | 54.5 (85) | 31.4 (49) |
| **II** | **4c** | 65.6 (394) | 21.3 (128) | 13.1 (79) |
|  | **6c** | 72.2 (340) | 16.8 (79) | 11.0 (52) |
| **III** | **4c** | 67.2 (338) | 19.9 (100) | 12.9 (65) |
|  | **6c** | 71.3 (181) | 17.3 (44) | 11.4 (29) |
| **IV** | **4c** | 71.5 (568) | 14.7 (117) | 13.8 (110) |
|  | **6c** | 69.2 (428) | 18.0 (111) | 12.8 (79) |
| **V** | **4c** | 53.7 (288) | 27.7 (149) | 18.6 (100) |
|  | **6c** | 53.3 (113) | 27.8 (59) | 18.9 (40) |
| **VI** | **4c** | 65.5 (569) | 17.0 (148) | 17.5 (152) |
|  | **6c** | 66.4 (1021) | 24.4 (375) | 9.2 (141) |
| **I-VI** | **4c** | 61.9 (2190) | 21.7 (770) | 16.4 (582) |
|  | **6c** | 64.8 (2105) | 23.2 (753) | 12.0 (390) |

**Supplementary Table 2.** Percentages of cells identified as neurons, glia and vascular cells in the mouse S1HL, fixated by immersion with no post-mortem delay, per individual animal. Data in parentheses refer to absolute numbers of cells identified.

**Supplementary Table 3.** Proportion of cells identified as neurons, glia and vascular cells in the mouse S1HL, fixated by immersion with 5 hours of post-mortem delay. Values in parentheses refer to the percentage of cells identified. Values for all layers (I–VI) correspond to the accumulated value. SD: Standard Deviation

| **Layer** | **Neuron (mean ± SD)** | **Glia (mean ± SD)** | **Vascular (mean ± SD)** |
| --- | --- | --- | --- |
| **I** | 0.14 ± 0.05 (14%) | 0.49 ± 0.04 (49%) | 0.37 ± 0.00 (37%) |
| **II** | 0.73 ± 0.01 (73%) | 0.16 ± 0.00 (16%) | 0.11 ± 0.01 (11%) |
| **III** | 0.66 ± 0.01 (66%) | 0.18 ± 0.01 (18%) | 0.16 ± 0.00 (16%) |
| **IV** | 0.74 ± 0.01 (74%) | 0.16 ± 0.01 (16%) | 0.10 ± 0.00 (10%) |
| **V** | 0.52 ± 0.01 (52%) | 0.29 ± 0.02 (29%) | 0.19 ± 0.02 (19%) |
| **VI** | 0.70 ± 0.02 (70%) | 0.22 ± 0.02 (22%) | 0.08 ± 0.00 (8%) |
| **I–VI** | 0.63 ± 0.01 (63%) | 0.23 ± 0.01 (23%) | 0.14 ± 0.01 (14%) |

|  |  | **Neuron (%)** | **Glia (%)** | **Vascular (%)** |
| --- | --- | --- | --- | --- |
| **I** | **ID3** | 10.9 (27) | 52.4 (130) | 36.7 (91) |
|  | **ID4** | 17.2 (43) | 46.4 (116) | 36.4 (91) |
| **II** | **ID3** | 73.1 (410) | 16.6 (93) | 10.3 (58) |
|  | **ID4** | 72.3 (300) | 16.4 (68) | 11.3 (47) |
| **III** | **ID3** | 66.8 (618) | 17.6 (163) | 15.6 (144) |
|  | **ID4** | 65.8 (351) | 18.5 (99) | 15.7 (84) |
| **IV** | **ID3** | 73.0 (548) | 17.0 (128) | 10.0 (75) |
|  | **ID4** | 74.4 (669) | 15.3 (138) | 10.3 (93) |
| **V** | **ID3** | 52.4 (522) | 27.1 (270) | 20.5 (204) |
|  | **ID4** | 51.4 (372) | 30.5 (221) | 18.1 (131) |
| **VI** | **ID3** | 68.1 (822) | 23.7 (286) | 8.2 (99) |
|  | **ID4** | 71.0 (641) | 20.3 (183) | 8.7 (79) |
| **I-VI** | **ID3** | 62.9 (2947) | 22.8 (1070) | 14.3 (671) |
|  | **ID4** | 63.8 (2376) | 22.1 (825) | 14.1 (525) |

**Supplementary Table 4.** Percentages of cells identified as neurons, glia and vascular cells in the mouse S1HL, fixated by immersion with 5 hours of post-mortem delay, per individual animal. Data in parentheses refer to absolute numbers of cells identified.

**Supplementary Table 5.** Proportion of cells identified as neurons and non-neurons in the mouse S1HL, fixated by perfusion. Values in parentheses refer to the percentage of cells identified. Values for all layers (I–VI) correspond to the accumulated value. SD: Standard Deviation

| **Layer** | **Neuron (mean ± SD)** | **Non-Neuron (mean ± SD)** |
| --- | --- | --- |
| **I** | 0.20 ± 0.02 (20%) | 0.80 ± 0.02 (80%) |
| **II** | 0.70 ± 0.03 (70%) | 0.30 ± 0.03 (30%) |
| **III** | 0.64 ± 0.07 (64%) | 0.36 ± 0.07 (36%) |
| **IV** | 0.71 ± 0.06 (71%) | 0.29 ± 0.06 (29%) |
| **V** | 0.50 ± 0.02 (50%) | 0.50 ± 0.02 (50%) |
| **VI** | 0.69 ± 0.02 (69%) | 0.31 ± 0.02 (31%) |
| **I–VI** | 0.61 ± 0.05 (61%) | 0.39 ± 0.05 (39%) |

**Supplementary Table 6.** Percentages of cells identified as neurons and non-neurons in the mouse S1HL, fixated by perfusion, per individual animal. Data in parentheses refer to absolute numbers of cells identified.

|  |  | **Neuron(%)** | **Non-Neuron (%)** |
| --- | --- | --- | --- |
| **I** | **ID41** | 20.9 (92) | 79.1 (349) |
|  | **ID42** | 18.7 (127) | 81.3 (552) |
| **II** | **ID41** | 72.5 (531) | 27.5 (201) |
|  | **ID42** | 68.4 (513) | 31.6 (237) |
| **III** | **ID41** | 68.9 (779) | 31.1 (352) |
|  | **ID42** | 59.1 (561) | 40.9 (389) |
| **IV** | **ID41** | 74.9 (832) | 25.1 (279) |
|  | **ID42** | 67.0 (649) | 33.0 (320) |
| **V** | **ID41** | 50.8 (334) | 49.2 (323) |
|  | **ID42** | 48.8 (280) | 51.2 (294) |
| **VI** | **ID41** | 70.7 (650) | 29.3 (269) |
|  | **ID42** | 67.8 (601) | 32.2 (294) |
| **I-VI** | **ID41** | 64.5 (3218) | 35.5 (1773) |
|  | **ID42** | 56.8 (2731) | 43.2 (2077) |

| **Comparison** | **Odds ratio** | **SE** | **Df** | **Null** | **Z ratio** | **p value** |
| --- | --- | --- | --- | --- | --- | --- |
| I / II | 0.075 | 0.012 | inf | 1 | -16.239 | <0.0001 |
| I / III | 0.075 | 0.012 | inf | 1 | -15.717 | <0.0001 |
| I / IV | 0.068 | 0.011 | inf | 1 | -17.148 | <0.0001 |
| I / V | 0.141 | 0.023 | inf | 1 | -12.023 | <0.0001 |
| I / VI | 0.084 | 0.013 | inf | 1 | -16.373 | <0.0001 |
| II / III | 0.996 | 0.102 | inf | 1 | -0.041 | 1.0000 |
| II / IV | 0.909 | 0.08 | inf | 1 | -1.083 | 0.8882 |
| II / V | 1.885 | 0.185 | inf | 1 | 6.438 | <0.0001 |
| II / VI | 1.114 | 0.088 | inf | 1 | 1.379 | 0.7396 |
| III / IV | 0.913 | 0.089 | inf | 1 | -0.932 | 0.9382 |
| III / V | 1.892 | 0.203 | inf | 1 | 5.949 | <0.0001 |
| III / VI | 1.119 | 0.1 | inf | 1 | 1.26 | 0.8069 |
| IV / V | 2.073 | 0.194 | inf | 1 | 7.784 | <0.0001 |
| IV / VI | 1.226 | 0.089 | inf | 1 | 2.808 | 0.0562 |
| V / VI | 0.591 | 0.05 | inf | 1 | -6.182 | <0.0001 |

**Supplementary Table 7.** GLMM analysis with Turkey post-hoc test for multiple layer comparison in the mouse S1HL, fixated by immersion with 0 hours of post-mortem delay. SE: Standard Error; Df: Degrees of Freedom.

| **Comparison** | **Odds ratio** | **SE** | **Df** | **Null** | **Z ratio** | **p value** |
| --- | --- | --- | --- | --- | --- | --- |
| I / II | 0.061 | 0.009 | inf | 1 | -18.917 | <0.0001 |
| I / III | 0.083 | 0.012 | inf | 1 | -17.761 | <0.0001 |
| I / IV | 0.058 | 0.008 | inf | 1 | -20.222 | <0.0001 |
| I / V | 0.151 | 0.021 | inf | 1 | -13.727 | <0.0001 |
| I / VI | 0.072 | 0.01 | inf | 1 | -19.13 | <0.0001 |
| II / III | 1.35 | 0.123 | inf | 1 | 3.304 | 0.0123 |
| II / IV | 0.952 | 0.087 | inf | 1 | -0.542 | 0.9944 |
| II / V | 2.466 | 0.214 | inf | 1 | 10.425 | <0.0001 |
| II / VI | 1.18 | 0.102 | inf | 1 | 1.929 | 0.3845 |
| III / IV | 0.705 | 0.056 | inf | 1 | -4.436 | 0.0001 |
| III / V | 1.827 | 0.134 | inf | 1 | 8.201 | <0.0001 |
| III / VI | 0.875 | 0.064 | inf | 1 | -1.841 | 0.4393 |
| IV / V | 2.591 | 0.191 | inf | 1 | 12.889 | <0.0001 |
| IV / VI | 1.24 | 0.091 | inf | 1 | 2.941 | 0.0385 |
| V / VI | 0.479 | 0.032 | inf | 1 | -10.913 | <0.0001 |

**Supplementary Table 8.** GLMM analysis with Turkey post-hoc test for multiple layer comparison in the mouse S1HL, fixated by immersion with 5 hours of post-mortem delay. SE: Standard Error; Df: Degrees of Freedom.

| **Comparison** | **Odds ratio** | **SE** | **Df** | **Null** | **Z ratio** | **p value** |
| --- | --- | --- | --- | --- | --- | --- |
| I / II | 0.104 | 0.01 | inf | 1 | -23.965 | <0.0001 |
| I / III | 0.138 | 0.012 | inf | 1 | -22.372 | <0.0001 |
| I / IV | 0.101 | 0.009 | inf | 1 | -25.555 | <0.0001 |
| I / V | 0.251 | 0.024 | inf | 1 | -14.571 | <0.0001 |
| I / VI | 0.11 | 0.01 | inf | 1 | -24.229 | <0.0001 |
| II / III | 1.335 | 0.098 | inf | 1 | 3.948 | 0.0011 |
| II / IV | 0.973 | 0.073 | inf | 1 | -0.361 | 0.9992 |
| II / V | 2.426 | 0.196 | inf | 1 | 10.974 | <0.0001 |
| II / VI | 1.06 | 0.081 | inf | 1 | 0.756 | 0.9746 |
| III / IV | 0.729 | 0.049 | inf | 1 | -4.735 | <0.0001 |
| III / V | 1.817 | 0.133 | inf | 1 | 8.153 | <0.0001 |
| III / VI | 0.794 | 0.055 | inf | 1 | -3.365 | 0.01 |
| IV / V | 2.492 | 0.187 | inf | 1 | 12.187 | <0.0001 |
| IV / VI | 1.089 | 0.077 | inf | 1 | 1.205 | 0.8345 |
| V / VI | 0.437 | 0.033 | inf | 1 | -10.803 | <0.0001 |

**Supplementary Table 9.** GLMM analysis with Turkey post-hoc test for multiple layer comparison in the mouse S1HL, fixated by perfusion. SE: Standard Error; Df: Degrees of Freedom.

|  |  | **Non-Neuron /Neuron Ratio** | **Glia/Neuron Ratio** |
| --- | --- | --- | --- |
| **I** | **4c** | 6.2 | 3.9 |
|  | **6c** | 6.1 | 3.9 |
| **II** | **4c** | 0.5 | 0.3 |
|  | **6c** | 0.4 | 0.2 |
| **III** | **4c** | 0.5 | 0.3 |
|  | **6c** | 0.4 | 0.2 |
| **IV** | **4c** | 0.4 | 0.2 |
|  | **6c** | 0.4 | 0.3 |
| **V** | **4c** | 0.9 | 0.5 |
|  | **6c** | 0.9 | 0.5 |
| **VI** | **4c** | 0.5 | 0.3 |
|  | **6c** | 0.5 | 0.4 |
| **I-VI** | **4c** | 0.6 | 0.4 |
|  | **6c** | 0.5 | 0.4 |

**Supplementary Table 10.** Estimated ratio of Non-neurons per Neuron and Glia per Neuron in each cortical layer in the mouse S1HL, fixated by immersion with 0 hours of post-mortem delay, per individual animal.

|  |  | **Non-Neuron /Neuron Ratio** | **Glia/Neuron Ratio** |
| --- | --- | --- | --- |
| **I** | **ID3** | 8.2 | 4.8 |
|  | **ID4** | 4.8 | 2.7 |
| **II** | **ID3** | 0.4 | 0.2 |
|  | **ID4** | 0.4 | 0.2 |
| **III** | **ID3** | 0.5 | 0.3 |
|  | **ID4** | 0.5 | 0.3 |
| **IV** | **ID3** | 0.4 | 0.2 |
|  | **ID4** | 0.3 | 0.2 |
| **V** | **ID3** | 0.9 | 0.5 |
|  | **ID4** | 0.9 | 0.6 |
| **VI** | **ID3** | 0.5 | 0.3 |
|  | **ID4** | 0.4 | 0.3 |
| **I-VI** | **ID3** | 0.6 | 0.4 |
|  | **ID4** | 0.6 | 0.3 |

**Supplementary Table 11.** Estimated ratio of Non-neurons per Neuron and Glia per Neuron in each cortical layer in the mouse S1HL, fixated by immersion with 5 hours of post-mortem delay, per individual animal.

|  |  | **Non-Neuron /Neuron Ratio** |
| --- | --- | --- |
| **I** | **ID41** | 3.8 |
|  | **ID42** | 4.3 |
| **II** | **ID41** | 0.4 |
|  | **ID42** | 0.5 |
| **III** | **ID41** | 0.5 |
|  | **ID42** | 0.7 |
| **IV** | **ID41** | 0.3 |
|  | **ID42** | 0.5 |
| **V** | **ID41** | 1.0 |
|  | **ID42** | 1.1 |
| **VI** | **ID41** | 0.4 |
|  | **ID42** | 0.5 |
| **I-VI** | **ID41** | 0.6 |
|  | **ID42** | 0.8 |

**Supplementary Table 12.** Estimated ratio of Non-neurons per Neuron in each cortical layer in mouse S1HL, fixated by perfusion, per individual animal.

**Supplementary Table 13.** Percentages of cells identified as neurons, glia and vascular cells in the human BA21, per individual case. Data in parentheses refer to absolute numbers of cells identified.

|  |  | **Neuron (%)** | **Glia (%)** | **Vascular (%)** |
| --- | --- | --- | --- | --- |
| **I** | **AB2** | 13.6 (58) | 60.2 (257) | 26.2 (112) |
|  | **AB3** | 9.9 (36) | 50.5 (184) | 39.6 (144) |
|  | **AB7** | 10.9 (52) | 57.2 (272) | 31.9 (152) |
| **II** | **AB2** | 55.1 (411) | 26.9 (201) | 18.0 (134) |
|  | **AB3** | 54.4 (260) | 26.4 (126) | 19.2 (92) |
|  | **AB7** | 55.8 (260) | 29.4 (137) | 14.8 (69) |
| **IIIa** | **AB2** | 42.6 (387) | 29.6 (269) | 27.8 (253) |
|  | **AB3** | 35.0 (131) | 35.9 (134) | 29.1 (109) |
|  | **AB7** | 39.1 (210) | 39.9 (214) | 21.0 (113) |
| **IIIb** | **AB2** | 25.4 (251) | 40.3 (399) | 34.3 (340) |
|  | **AB3** | 28.1 (190) | 41.2 (279) | 30.7 (208) |
|  | **AB7** | 30.7 (188) | 33.1 (203) | 36.2 (222) |
| **IV** | **AB2** | 42.6 (276) | 36.6 (237) | 20.8 (135) |
|  | **AB3** | 40.7 (100) | 48.3 (119) | 11.0 (27) |
|  | **AB7** | 36.7 (242) | 60.3 (397) | 3.0 (20) |
| **V** | **AB2** | 29.2 (331) | 41.3 (468) | 29.5 (335) |
|  | **AB3** | 28.3 (109) | 47.3 (182) | 24.4 (94) |
|  | **AB7** | 22.6 (378) | 55.8 (931) | 21.6 (360) |
| **VI** | **AB2** | 16.8 (136) | 56.3 (456) | 26.9 (218) |
|  | **AB3** | 16.7 (37) | 57.6 (128) | 25.7 (57) |
|  | **AB7** | 14.3 (92) | 63.6 (409) | 22.1 (142) |
| **I-VI** | **AB2** | 32.7 (1850) | 40.3 (2287) | 27.0 (1527) |
|  | **AB3** | 31.4 (863) | 42.0 (1152) | 26.6 (731) |
|  | **AB7** | 28.1 (1422) | 50.6 (2563) | 21.3 (1078) |

| **Comparison** | **Odds ratio** | **SE** | **Df** | **Null** | **Z ratio** | **p value** |
| --- | --- | --- | --- | --- | --- | --- |
| I / II | 0.107 | 0.011 | inf | 1 | -22.166 | <0.0001 |
| I / IIIa | 0.198 | 0.02 | inf | 1 | -16.146 | <0.0001 |
| I / IIIb | 0.345 | 0.034 | inf | 1 | -10.658 | <0.0001 |
| I / IV | 0.198 | 0.02 | inf | 1 | -15.862 | <0.0001 |
| I / V | 0.376 | 0.036 | inf | 1 | -10.081 | <0.0001 |
| I / VI | 0.699 | 0.077 | inf | 1 | -3.232 | 0.021 |
| II / IIIa | 1.846 | 0.126 | inf | 1 | 8.945 | <0.0001 |
| II / IIIb | 3.221 | 0.218 | inf | 1 | 17.266 | <0.0001 |
| II / IV | 1.843 | 0.132 | inf | 1 | 8.547 | <0.0001 |
| II / V | 3.505 | 0.225 | inf | 1 | 19.511 | <0.0001 |
| II / VI | 6.524 | 0.542 | inf | 1 | 22.558 | <0.0001 |
| IIIa / IIIb | 1.745 | 0.117 | inf | 1 | 8.301 | <0.0001 |
| IIIa / IV | 0.999 | 0.071 | inf | 1 | -0.019 | 1.0000 |
| IIIa / V | 1.899 | 0.12 | inf | 1 | 10.11 | <0.0001 |
| IIIa / VI | 3.534 | 0.291 | inf | 1 | 15.325 | <0.0001 |
| IIIb / IV | 0.572 | 0.04 | inf | 1 | -7.949 | <0.0001 |
| IIIb / V | 1.088 | 0.068 | inf | 1 | 1.343 | 0.8318 |
| IIIb / VI | 2.025 | 0.166 | inf | 1 | 8.607 | <0.0001 |
| IV / V | 1.901 | 0.125 | inf | 1 | 9.743 | <0.0001 |
| IV / VI | 3.539 | 0.3 | inf | 1 | 14.91 | <0.0001 |
| V / VI | 1.861 | 0.146 | inf | 1 | 7.906 | <0.0001 |

**Supplementary Table 14.** GLMM analysis with Turkey post-hoc test for multiple layer comparison in human BA21. SE: Standard Error; Df: Degrees of Freedom.

**Supplementary Table 15.** Estimated ratio of Non-neurons per Neuron and Glia per Neuron in each cortical layer of Human BA21, per individual case.

|  |  | **Non-Neuron/Neuron Ratio** | **Glia/Neuron Ratio** |
| --- | --- | --- | --- |
| **I** | **AB2** | 6.4 | 4.4 |
|  | **AB3** | 9.1 | 5.1 |
|  | **AB7** | 8.2 | 5.2 |
| **II** | **AB2** | 0.8 | 0.5 |
|  | **AB3** | 0.8 | 0.5 |
|  | **AB7** | 0.8 | 0.5 |
| **IIIa** | **AB2** | 1.3 | 0.7 |
|  | **AB3** | 1.9 | 1.0 |
|  | **AB7** | 1.6 | 1.0 |
| **IIIb** | **AB2** | 2.9 | 1.6 |
|  | **AB3** | 2.6 | 1.5 |
|  | **AB7** | 2.3 | 1.1 |
| **IV** | **AB2** | 1.3 | 0.9 |
|  | **AB3** | 1.5 | 1.2 |
|  | **AB7** | 1.7 | 1.6 |
| **V** | **AB2** | 2.4 | 1.4 |
|  | **AB3** | 2.5 | 1.7 |
|  | **AB7** | 3.4 | 2.5 |
| **VI** | **AB2** | 5.0 | 3.4 |
|  | **AB3** | 5.0 | 3.5 |
|  | **AB7** | 6.0 | 4.4 |
| **I-VI** | **AB2** | 2.1 | 1.2 |
|  | **AB3** | 2.2 | 1.3 |
|  | **AB7** | 2.6 | 1.8 |

**Video 1. Visualization of vascular cells as revealed by their distinct cylindrical shape and dense, clustered distribution in the DAPI channel through the Z-axis**. Note the absence of these cells in the NeuN channel, validating that they are not neurons.
